# Protein Metabolism Changes and Alterations in Behavior of Trace Amine-Associated Receptor 1 Knockout Mice Fed a High-Fructose Diet

**DOI:** 10.3390/neurolint15010022

**Published:** 2023-02-28

**Authors:** Sergey A. Apryatin, Ilya S. Zhukov, Ekaterina A. Zolotoverkhaya, Saveliy R. Kuvarzin, Temirkan A. Khunagov, Sanelya V. Ushmugina, Victor M. Klimenko

**Affiliations:** 1 Institute of Experimental Medicine, 197376 Saint Petersburg, Russia; 2 Institute of Translational Biomedicine, Saint Petersburg State University, 199034 Saint Petersburg, Russia; 3 Golikov Research Center of Toxicology, 193019 Saint Petersburg, Russia; 4 Faculty of Biology, Lomonosov Moscow State University, 119991 Moscow, Russia; 5 Department of Medical and Biological Disciplines, Moscow Medical University Reaviz, 107564 Moscow, Russia

**Keywords:** trace amine-associated receptor 1, TAAR1, dopamine, depression, high-fructose diet, G protein-coupled receptors, energy metabolism, catabolism, liver, depression ratio, AST/ALT ratio

## Abstract

Trace amines and their receptors are a family of G protein-coupled receptors widely distributed in the central nervous system and periphery. The trace amine-associated receptor 1 (TAAR1) plays a significant role as a therapeutic target for schizophrenia, depression, diabetes, and obesity. In this study, TAAR1 knockout mice and WT groups were tested in conditions of a high-fructose diet. The consumption of a high-fructose diet may be due to the influence on the metabolism processes by dopamine in the brain, neuromotor function, and level of anxiety of TAAR1 knockout mice. During a comparative analysis of behavioral, biochemical, and morphological parameters, significant differences were found between liver and biochemical parameters, the regulation of protein metabolism (AST/ALT ratio, creatine kinase activity, urea), and alterations in behavior. An elevated plus maze analysis showed the influence of fructose and genetic factors on the level of anxiety. A new marker of the grooming microstructure (depression ratio) was tested, which showed high efficiency as a marker of depression-like behavioral changes and a possible association with dopamine-dependent regulation of protein metabolism. These results confirm a possible association of the *TAAR1* gene knockout with an increase in catabolic reaction levels by AST/ALT-dependent and possible dopamine-mediated protein metabolism regulation and depression-like behavior.

## 1. Introduction

Trace amines (TA) and their receptors are emerging as important regulators of complex forms of behavioral disorders [1,2]. The term ‘trace amine’ was coined and introduced into scientific practice in the early 1970s by Alan Boulton [3], who wanted to emphasize the very low (less than 100 ng/g tissue) concentration of trace amines compared to that of classical neurotransmitters [4]. The first trace amine-associated receptor (TAAR1) was discovered in 2001 [5,6], and this led to the understanding of the functional role of a separate group of endogenous monoamines with their own independent receptor system involved in the pathogenesis of various diseases [2]. TAAR1 is a G protein-coupled receptor (GPCR) that plays an important role in the regulation of dopaminergic, serotonergic, and glutamatergic activity [7,8,9].

TA, such as β-phenylethylamine, p-tyramine, and p-octopamine, are best known as the result of amino acid decarboxylation, i.e., the thermal or enzymatic processing of food, both with the participation of the microflora in the gastrointestinal tract [2,10] and without it (meat, fish, cocoa, chocolate, cheese, etc.). They are also produced endogenously in mammalian tissues and include also metabolites of endogenous monoamine neurotransmitters, such as dopamine, serotonin, and norepinephrine [8,9,10]. The system of trace amines affects dopamine and other systems regulating the development of psychiatric, neurodegenerative, and metabolic disorders by affecting signaling, neurogenesis, energy metabolism, and other physiological processes [2,11,12,13,14,15,16,17].

Trace amines are found not only in thermally or enzymatically processed foods but also in many fresh foods in concentrations in the range of milligrams per kilogram [2,15]. Many biogenic amines, such as tyramine, are present in nanomolar concentrations in blood plasma and in the central nervous system (mainly in neurons) of healthy people [1]. Over the past few years, there has been an increase in promising research on the trace amine system in biomedicine, including the development of preclinical and clinical studies of drugs, cosmetics, dietary supplements, and specialty foods. Research has also established the role of TA in the control of behavior, energy metabolism, and cellular immune responses, including interactions with the microbiota in the biochemical transformations of nutrients in the body and, therefore, in the pathogenesis of alimentary-dependent diseases [2,17].

There is now a significant amount of scientific information relating to the influence of dopamine systems on behavioral and metabolic disorders, which has made it possible to partially discover some of the mechanisms involved in the development of the above pathological conditions, including interaction with reward systems, dopamine signal reception, mood regulation, and metabolic changes [18,19,20,21,22,23,24,25,26]. The connection of the trace amine system with dopamine and other monoamine systems of the brain has been established, but these studies only began around 20 years ago, i.e., when the discovery of the first trace amine receptor, TAAR1, and other TAARs (TAAR2-TAAR9) was made [2,5,27]. Drugs based on TAAR1 receptor agonism are now being developed for the treatment of schizophrenia and other mental disorders [28,29,30,31].

TAAR1 is the receptor that is best studied in the TAAR family [2,17]. Its expression has been shown in nervous tissue (glial cells [32] and neurons [33]), as well as in other organs and tissues, for example, in the gut, stomach, and pancreas [34]. The mechanisms of TAAR1 receptor activation are associated with intracellular signaling via cAMP, phosphorylation protein kinase A, and subsequent signal transduction into the nucleus [34]. In the striatum of TAAR1 knockout (TAAR1-KO) mice, overexpression of both mRNA and D2-dopamine receptor protein was found, but no changes were noted in the density of D1 dopamine receptors. Furthermore, the AKT/GSK3 signaling pathway (not associated with the G protein-mediated D2 dopamine receptor signaling) was selectively activated, which is associated with the phosphorylation of AKT and GSK3β [21]. TAAR1 is being studied as a potential therapeutic target in the treatment of various mental disorders, such as schizophrenia [2,27,35,36,37,38,39,40,41,42,43,44,45,46]. Ulotaront (SEP-363856) is a TAAR1 agonist with 5-HT1A receptor agonist activity, currently being tested in phase III clinical development, with very promising results from the phase II trials, which led to the FDA designation as a breakthrough therapy for the treatment of schizophrenia [26,27,28,29,30,40,47,48,49,50,51,52].

The latest hematological and biochemical studies demonstrate the importance of the TAAR receptor family in the periphery. It was found that deletion of the TAAR5 receptor gene leads to erythrocyte fragility changes [53]. Furthermore, TAAR9 knockout rats demonstrated decreased low-density lipoprotein cholesterol levels in comparison to control rats [54]. However, when biochemical and hematological parameters were analyzed in TAAR1 knockout (TAAR1-KO) mice, only the creatine kinase levels were found to be minimally changed in TAAR1-KO male mice [55]. Thus, we speculated that the high fructose diet may potentially reveal specific metabolism changes in the TAAR1-KO mice.

Expression of TAAR1 in the brain of experimental animals is observed in monoaminergic, in particular dopaminergic, neurons [1,7,21]. In addition, TAAR1 activation by a selective small molecule agonist increased glucose-dependent insulin secretion in INS1E cells and human islets and elevated plasma peptide YY (PYY) levels in mice. In diabetic mice (db/db line), the TAAR1 agonist normalized glucose excursion during an oral glucose tolerance test, reduced food intake and body weight, and insulin sensitivity was improved [35]. It was shown, also, that selective TAAR1 agonists induced conditioned taste aversion [56] and reduced binge eating [23]. TAAR1 activation has separately been reported to regulate the secretion of the closely related PYY, and ketonuria was associated with a loss of body fat due to a switch from glucose to lipid metabolism [57,58]. Moreover, the consumption of fructose significantly modifies the intensity and direction of these effects, which may presumably be due to the influence of this diet on the processes of dopamine metabolism in the brain, neuromotor function, and level of anxiety [59].

TAAR1 agonists can play a certain role in alleviating depression-like and anxiety-like behaviors in animal models [27,28,47,49]. Another report showed that SEP-363856 and duloxetine could have a significant antidepressant effect in a mouse forced swim test. The results also indicated that Ulotaront can demonstrate antidepressant-like effects in mice, according to other behavioral tests (tail suspension and sucrose preference tests) [48]. The specific mechanism of action has yet to be explored. However, many biological mechanisms of the monoamine systems that influence behavioral and metabolic disorders are currently unknown or insufficiently studied [2,19]. Therefore, the search for new models of behavioral and metabolic disorders associated with dopamine systems, trace amines, and their receptors, is a priority.

Biochemical and integral indicators in the blood are often used to assess metabolic changes. Among the biochemical indicators, one of the most valuable is protein metabolism, including total protein, urea, and transaminase activity (AST, ALT, and De Ritis ratio). The aspartate aminotransferase (AST) activity plays an important role in the regulation of catabolic reactions of the body, and alanine aminotransferase (ALT)–anabolic reactions, which prove that these biochemical indicators are not only the result of the cytolysis of myocardial and liver cells, respectively [55,59].

The level of locomotor activity is usually studied using the open field test. The anxiety level was assessed using the elevated plus maze (EPM) test, which allows for the assessment of the severity of the emotional reaction of fear and anxiety, motor activity, the speed of orienting reactions, and other behavioral changes [18,55,58,59]. One of the important tasks of the study was the search for new behavioral indicators to assess the depression-like behavior of TAAP1-KO mice, based on the microstructure of grooming [60,61]. Overall, the aim of our research work is to study the effect of the *TAAR1* gene knockout on the regulation of energy metabolism and depression-like behavior in mice fed a diet with excess fructose.

## 2. Materials and Methods

### 2.1. Study Design

All the animal studies were carried out according to the guidelines of the Ministry of Health of the Russian Federation, FELASA, and RusLASA. All the experiments were approved by the Saint Petersburg State University Ethical Committee for Animal Research (No. 131-03-1, 16 July 2020). Wild-type (WT) and TAAR1-KO mice were derived by crossing (over 20 generations) heterozygous TAAR1 C57BL6/129SvJ animals. The experiment was carried out on TAAR1-KO female knockout mice aged 2–3 months, genotyped before and after the experiment. The mice (N = 24) were divided into four groups (N = 6): TAAR1-KO homozygotes and WT, each of which was divided into two more groups, one of which received a standard control diet (WT and TAAR1-KO), and the other received a control diet with the addition of a 20% fructose solution instead of water (WT^d^ and TAAR1-KO^d^) for 62 days.

The body mass of the mice was measured weekly. After decapitation, the relative masses of the internal organs were determined, blood plasma was taken [55], and the key biochemical parameters and morphological changes in the liver were evaluated.

### 2.2. Behavioral Tests

#### 2.2.1. Open Field Test (OF)

An open field test (OF) was used to measure locomotor and exploratory activity. The apparatus for the test consisted of a gray plastic round arena (diameter, 60 cm). The mice were placed at the center of the arena, and spontaneous exploration activity, grooming microstructure, and locomotor activity were recorded manually for five minutes per mouse.

#### 2.2.2. Elevated Plus Maze Test (EPM)

The animals’ anxiety levels were evaluated using an elevated plus maze (EPM) test, which was a plus maze raised above the floor with two open (OA) and two closed arms (CA). The EPM test is based on the natural mice behavior of preferring closed spaces and avoiding open ones. At the beginning of the experiment, the mice were placed in the center of the EPM. Their behavioral patterns (the number of grooming/hanging episodes and the total time in open/closed arms) were counted by an operator for five minutes per mouse, without any changes in light intensity. The OF and EPM tests were performed on the 56th and 59th day of the experiment, respectively.

#### 2.2.3. Grooming Microstructure Analysis

A grooming stage analysis was assessed according to the following (head to tail) system: no grooming (stage 0), paw licking (stage 1), nose and face washing (stage 2), head washing (stage 3), body grooming (stage 4), scratching the body (stage 5), washing the hind legs and tail (stage 6), washing the genitals (stage 7). The grooming microstructure indicators (latency in seconds) at the beginning of grooming (LG), total seconds spent on grooming (TGT), and the number of grooming acts (NGA) were noted from the video recording of the OF and EPM [60,61]. The grooming analysis also included the average duration of a single act of grooming (ADSAG) (defined as the ratio of TGT/NGA), the number of grooming stages (NGS), and the average duration of one stage (ADOSG = TGT/NGS). Approbation of the diagnostic significance of the new depression ratio (DR)—defined as the ratio of the total time spent on grooming to the latency of the start of grooming (TGT/LG)—was carried out by a grooming microstructure analysis (EPM test).

### 2.3. Measurement of Biochemical Parameters

A TAAR1 biochemical analysis was performed using a Random Access A-25 automatic analyzer (Biosystems S.A., Barselona, Spain), utilizing the spectrophotometer principle. The blood serum samples were stored at −20 °C before analysis. The following biochemical parameters were analyzed: alanine aminotransferase (ALT), aspartate aminotransferase (AST), total protein, urea, triglycerides (TG), lactate dehydrogenase (LDH), and creatine kinase. 

### 2.4. Histological Analysis

Histological wiring and staining of liver preparations were performed with eosin-hematoxylin, according to Van Gieson (connective tissue), and Sudan black (total lipids), according to standard methods [62,63].

### 2.5. Statistical Analysis

A two-way analysis of variance (ANOVA) with a post hoc Tukey HSD test was used to compare all the biochemical data and behavioral parameters. The analyses were performed using GraphPad Prism 8 for Windows (GraphPad Software, San Diego, CA, USA). The values of *p* < 0.05 were considered to be significant.

## 3. Results

**Figure 1:**
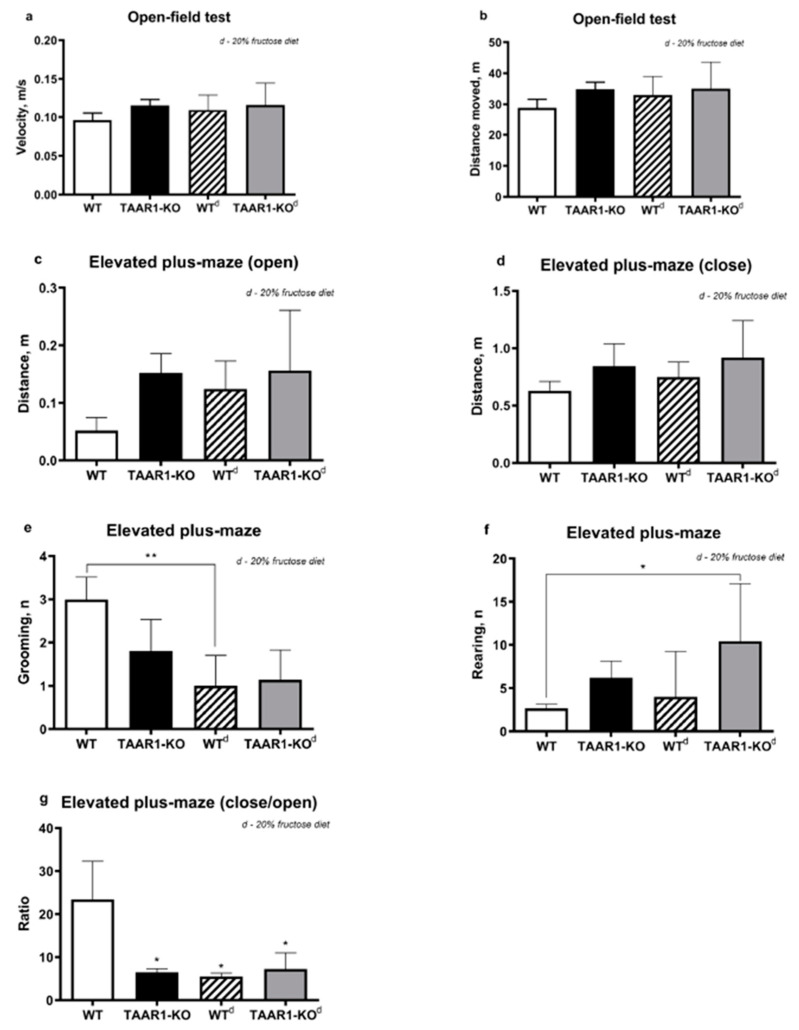
Open-field and elevated plus maze test results. Comparative analysis of open-field and elevated plus maze tests demonstrates minimal alterations in behavioral parameters between TAAR1-KO and WT mice female groups (**a**–**g**). 20% fructose diet led to decreased grooming in WT group ((**e**), **—*p* < 0.001), increased rearing in TAAR1-KO^d^ group ((**f**) *—*p* < 0.05), and decreased EPM close/open ratio level of all groups compared to the WT group ((**g**) *—*p* < 0.05). Data are mean ± SEM.

These experiments were performed to investigate the effect of a high-fructose diet on TAAR1-KO mice. In the open field test, there were no correlational differences in locomotor activity between the WT and TAAR1-KO groups on both diets (Figure 1a,b). It also showed no effect of a high-fructose diet on the average speed and total distance in the OF test. The average distance covered by the mice in the TAAR1-KO group in the open arms of the maze in the EPM test was significantly higher than the WT control group (Figure 1c,d), which may indicate a decrease in the level of anxiety in the mice with knockout of the *TAAR1* gene. These data are also confirmed by the decrease in the values of the ratio CA/OA in all the studied groups compared with the control group (Figure 1g).

Interestingly, in the TAAR1-KO group, there was a trend toward an increase in the number of rearing acts in the EPM test, and, in the TAAR1-KO^d^ group, this difference was significant in relation to the WT control group (Figure 1f).

In the EPM test, a decrease in the number of grooming acts was recorded in the WT^d^ group compared to the control group, which presumably indicates the effect of fructose on the increase in anxiety levels (Figure 1e).

In the open field test, the traditional grooming parameters (latency in seconds) of the beginning of grooming (LG), total time (in seconds) spent on grooming (TGT), and the number of grooming acts (NGA) were significantly different in the TAAR1 knockouts and controls. The grooming latency in the TAAR1-KO mice was almost two times longer than in the control group (WT: 45 ± 12; TAAR1-KO: 117 ± 25, *p* = 0.0181). In addition, this group demonstrated significantly shorter grooming stages (WT: 7.8 ± 2.7; TAAR1-KO: 3.6 ± 1.2, *p* = 0.0345) and a sharp decrease in frequency (WT: 36 ± 16; TAAR1-KO: 6 ± 1, *p* = 0.0128) and duration of grooming (WT: 48 ± 13; TAAR1-KO: 20 ± 6, *p* = 0.0314). This result shows the reduced grooming and the intermittent nature of its stages. The values of the depression ratio (DR) in both groups were significantly different (WT: 0.67 ± 0.22; TAAR1-KO: 0.14 ± 0.02, *p* = 0.0297) in contrast to the average number of acts and stages of grooming in both groups (data not shown). A decrease of the DR value indicates the development of depressive-like behavior in the TAAR1-KO mice. Thus, the grooming microstructure of the TAAR1-KO mice correlated with depressive-like behavioral changes, which is consistent with the current understanding of the phenotype of this knockout line.

A two-fold decrease in total specific energy consumption was found in the TAAR1-KO knockout mice on the high-fructose diet compared to those on the control diet.

**Figure 2:**
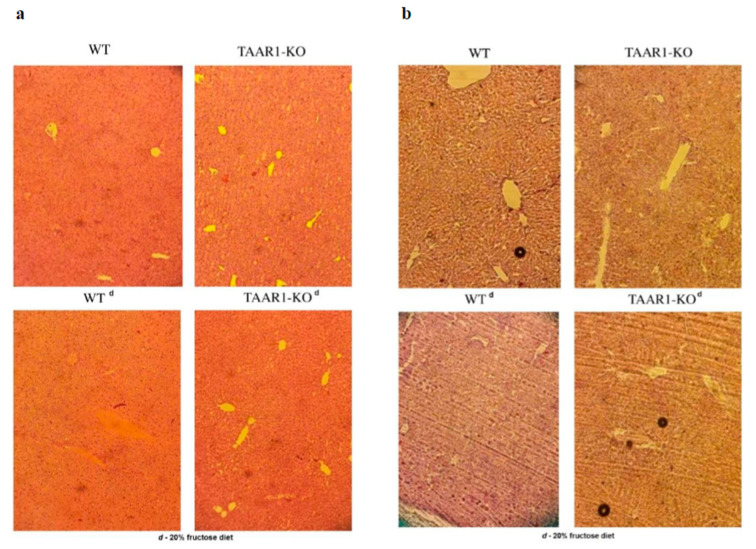
Morphological picture of the liver tissue of TAAR1-KO knockout mice on a 20% fructose diet. Staining with eosin-hematoxylin (**a**) and Sudan black (**b**) for total lipids. There are minimal alterations in liver tissue. Magnification 230×.

A morphological study of the liver showed no effect of the high-fructose diet and *TAAR1* gene knockout on lipid accumulation in the liver parenchyma (Figure 2).

**Figure 3:**
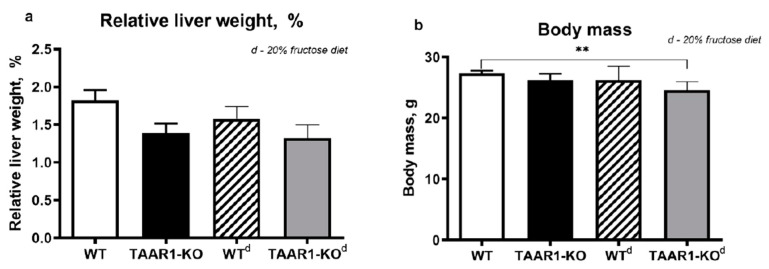
Relative liver weight (**a**) and body mass (**b**) of TAAR1-KO knockout mice fed a 20% fructose diet. There are minimal alterations in organ weight. **—*p* < 0.05. Data are mean ± SEM.

At the same time, in the TAAR1-KO^d^ group, the average body mass was lower than in the WT group (Figure 3b), but the relative mass of the liver was unchanged (Figure 3a).

However, in older animals (over eight months of age) with the TAAR1-KO genotype, there was often an increase in body weight compared to the wild type, which may indicate the influence of this receptor on metabolic processes at a more mature age.

**Figure 4:**
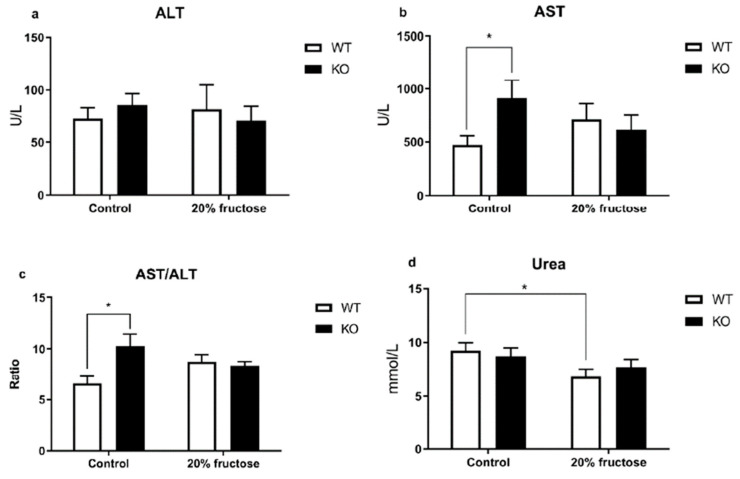

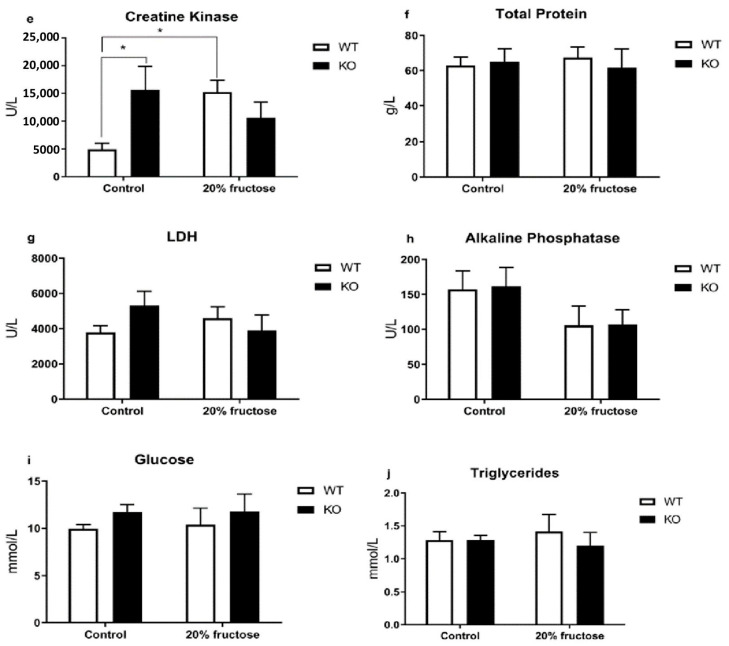
Comparative analysis of basic biochemical parameters in the blood of TAAR1-KO and WT female mice fed a 20% fructose diet. (**a**) Alanine aminotransferase (ALT), (**b**) aspartate aminotransferase (AST), (**c**) De Ritis ratio (AST/ALT), (**d**) urea, (**e**) creatine kinase, (**f**) total protein, (**g**) lactate dehydrogenase (LDH), (**h**) alkaline phosphatase (ALP), (**i**) glucose, (**j**) triglycerides. The biochemical screening reveals significant differences in several demonstrated parameters. Data are mean ± SEM. n = 5–6. *—*p* < 0.05.

The analysis of biochemical parameters revealed a number of changes in the indicators of the De Ritis ratio, the levels of AST (Figure 4b, *p* = 0.0286), creatine kinase (Figure 4e, *p* = 0.142, WT vs. KO; *p* = 0.0175, WT vs. WT^d^) activity, and the concentration of urea (Figure 4d) in the blood plasma.

The De Ritis ratio (AST/ALT) in the TAAR1-KO mice was unchanged regardless of the diet consumed, and there was a tendency for it to decrease in the WT^d^ mice in comparison with the WT group (Figure 4c, *p* = 0.0194). An increase in the AST activity and De Ritis ratio values in the TAAR1-KO compared with the control group may indicate an activation of catabolic reactions, as evidenced by high relative levels of creatine kinase in the TAAR1-KO group on the control diet compared to the WT, and high urea levels in the groups with a high-fructose diet.

## 4. Discussion

In this study, we demonstrated a possible association of the TAAR1 function with the increase in high fructose-induced catabolic reactions via AST/ALT-dependent processes and possible dopamine-mediated protein metabolism regulation and depression-like behavior.

There were no correlational differences in the locomotor activity between the WT and TAAR1-KO groups in the OF test. Neither was there any effect of the high-fructose diet on the average speed and total distance covered by the WT and TAAR1-KO mice in the OF test, and the number of transitions between the maze zones in the EPM test was shown in comparison with the control group. The average distance covered by the TAAR1-KO mice in the open arms of the maze in the EPM test was significantly higher than the WT control group, which may indicate a decrease in the anxiety of the TAAR1-KO mice. These data are also confirmed by the decrease in the ratio of CA/OA in all the studied groups in comparison with the control. The decrease in the number of grooming events in the WT^d^ group compared to the control group suggests that the fructose was increasing anxiety. It is interesting to note that the TAAR1-KO mice tended to increase the number of racks in the EPM test, and, in the fructose-supplemented group, this difference was significant relative to the WT control group.

The increase in AST activity and the values of the De Ritis coefficient (AST/ALT) in the blood of the TAAR1-KO mice compared with the WT control group may indicate an activation of catabolic reactions. These results complement the study on male TAAR1-KO mice, where the indicators of creatine kinase activity were the opposite, which can be explained by differences in the metabolism between female and male mice [55]. This finding is confirmed by the high relative levels of creatine kinase in the TAAR1-KO group on the control diet compared to the WT, and the high urea levels in groups with the high-fructose diet. These results confirm the association between the *TAAR1* gene and energy metabolism—particularly protein metabolism.

It was also found that the parameters of the grooming microstructure of the TAAR1-KO mice were correlated with depressive behavioral changes, which is consistent with modern ideas about this knockout line. The last studies demonstrated that *TAAR1* gene knockout in male mice leads to significantly decreased self-grooming activity and significant changes in grooming microstructure [64]. To assess the degree of depression, a depression ratio (DR) was proposed, and the average values of this decreased for the TAAR1-KO mice in comparison with the control group.

The average distance covered by the TAAR1-KO mice in the open arms of the maze in the EPM test was significantly higher than those in the WT^d^ control group, which may indicate a decrease in anxiety in mice with the *TAAR1* gene knockout. These data are also confirmed by the decrease in the values of the ratio CA/OA in all the studied groups in comparison with the control.

The decrease in the number of grooming events in the WT^d^ group compared to the control group suggests that the fructose was increasing their anxiety. It is interesting to note that, in the TAAR1-KO mice, there was a general increase in the number of racks in the EPM test and, in the group with a high-fructose diet, this difference was significant in relation to the WT^d^ control group.

The total specific energy consumption of the TAAR1 knockout mice on a high-fructose diet was two times lower compared to those on the control diet and did not depend on the genotype.

A morphological examination of the liver tissue did not reveal any differences between the TAAR1-KO mice and the WT mice in both the control and high-fructose diets. An increase in AST activity and the values of the De Ritis ratio (AST/ALT) in the blood of the TAAR1-KO mice compared with the control group may indicate the activation of catabolic reactions. This finding is confirmed by the high relative levels of creatine kinase in the TAAR1-KO group on the control diet compared to the WT, as well as the high urea levels in groups on a high-fructose diet (Figure 4b,c,e). The absence of changes in the De Ritis ratio in these groups may be another indirect confirmation of the co-expression of TAAR1 with the dopamine D2R receptors described previously [9,19]. This result points to a relationship of AST/ALT in dopamine systems through their important regulatory role in associated metabolic processes, with the consumption of high-calorie diets described in the author’s other study [59]. Thus, the increased AST and creatine kinase activity, AST/ALT ratio, and urea level in blood plasma showed a statistically significant increase in the TAAR1 knockout mice in comparison with the wild type, which may be the cause of the dopamine-mediated protein metabolism regulation and depression-like behavior.

The results confirm our hypothesis of a possible association of the *TAAR1* gene knockout with the increase in catabolic reaction levels by AST/ALT-dependent and possible dopamine-mediated protein metabolism regulation and depression-like behavior. The limitations and strengths are partly discussed in the discussion section. Unfortunately, the relationship between diet and *TAAR1* gene knockout has not been fully discribed. Therefore, further experiments are required to understand the problem.

## 5. Conclusions

We demonstrated here that the grooming microstructure in the TAAR1-KO knockout mice correlates with depressive behavioral changes. To assess the depression level, we introduced a DR (depression ratio), the average values of which decreased as compared to the reference group in the case of the TAAR1-KO knockout mice. In this study, the depression ratio was shown to be a marker of depression-like behavioral changes.

Our results confirm our hypothesis of a possible association of the *TAAR1* gene knockout with an increase in catabolic reaction levels by AST/ALT-dependent and possible dopamine-mediated protein metabolism regulation.

The results of this study confirm the relationship of the *TAAR1* gene with energy metabolism and, in particular, protein metabolism. The consequences of such studies may include the identification of new behavioral, biochemical, and other markers of functional disorders of monoamine systems and metabolic dysfunctions. There may also be a prospect of transferring the identified molecular markers to the preclinical and clinical treatment of these diseases, including differential diagnosis and preventive therapy.

## Data Availability

All data listed in this review article are publicly accessible on PubMed.
